# Application and Research Progress of Self-Assembling Protein Nanoparticles in Vaccine Development

**DOI:** 10.3390/ijms27104503

**Published:** 2026-05-18

**Authors:** Yue Zhang, Yi Ru, Xiuping Li, Guanghua Wang, Yong Hu, Yingna Jian, Liqing Ma

**Affiliations:** 1 Qinghai Provincial Key Laboratory of Pathogen Diagnosis for Animal Diseases and Green Technical Research for Prevention and Control, College of Agriculture and Animal Husbandry, Academy of Animal Sciences and Veterinary, Qinghai University, Xining 810016, China; 2 State Key Laboratory for Animal Disease Control and Prevention, Lanzhou Veterinary Research Institute, College of Veterinary Medicine, Lanzhou University, Chinese Academy of Agricultural Sciences, Lanzhou 730046, China

**Keywords:** self-assembling protein nanoparticles, virus-like particles, ferritin, artificially designed nanoparticles, vaccine development

## Abstract

This review systematically evaluates research on self-assembling protein nanoparticles (SPNPs)-based vaccine, focusing on three major categories: virus-like particles (VLPs), natural protein nanoparticles (e.g., ferritin and encapsulin), and computationally designed protein nanoparticles—with comparative analyses of their design strategies, immunogenicity, and applicability in next-generation vaccines. VLPs can elicit robust humoral and cellular immune responses due to their virus-mimetic structures. Natural protein nanoparticles provide excellent biocompatibility and controllable self-assembly for multivalent antigen presentation. Artificially designed protein nanoparticles allow precise structural optimization to facilitate tailored antigen display and immune modulation. Overall, SPNPs constitute a versatile and powerful platform for vaccine development. By integrating natural and engineered design principles, they offer new opportunities for rational vaccine design and the development of safer and more effective immunization strategies.

## 1. Introduction

In recent years, the rapid advancement of nanotechnology has significantly advanced the research of novel vaccine platforms, among which self-assembling protein nanoparticles (SPNPs) have emerged as a prominent focus of research due to their remarkable biological properties. Compared with nanocarriers constructed from metals (e.g., gold or silver nanoparticles), liposomes, or polymeric materials, naturally derived protein nanoparticles exhibit more pronounced advantages in biocompatibility, biodegradability, and immune modulation. Although metallic nanomaterials possess excellent physicochemical stability and controllable size, their potential cytotoxicity and difficulty in in vivo clearance limit their applications in biological systems [1]. Liposomes, while effective in antigen delivery, are prone to structural instability or degradation under environmental influences [2]. Biodegradable polymeric nanoparticles (e.g., Poly(lactic-co-glycolic acid), chitosan) enable controlled release and favorable pharmacokinetics but generally exhibit limited intrinsic immunogenicity, thereby necessitating co-delivery of adjuvants or immunostimulatory molecules to elicit robust humoral and cellular responses [3]. In contrast, not only possess regular three-dimensional structures, uniform size distribution, and engineerable surface modifications, but also can directly activate the immune system through their intrinsic molecular properties, effectively enhancing antigen immunogenicity and vaccine efficacy [4]. Furthermore, through genetic engineering and chemical modification, their surface structure and immunological performance can be further optimized, making them highly promising platforms for vaccine delivery and immune enhancement [5].

**Figure 1:**
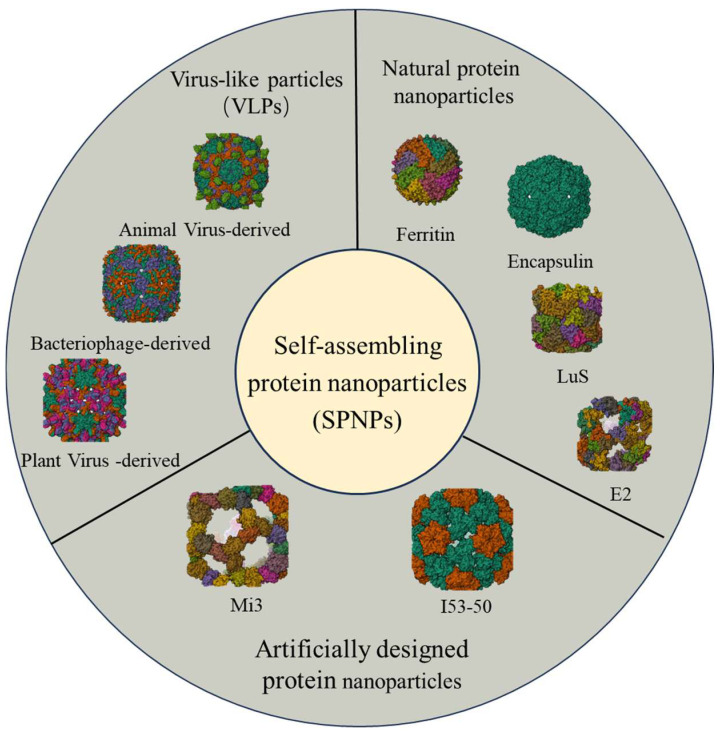
Schematic classification of protein nanoparticles. Protein nanoparticles can be broadly classified into three types: (1) VLPs; (2) natural protein nanoparticles; and (3) engineered or artificial protein nanoparticles.

SPNPs are formed through the spontaneous assembly of one or more protein molecules and typically exhibit hollow, cage-like structures with highly stable shells, demonstrating broad application prospects in biomedical research. Based on their origin and design strategies, SPNPs can be divided into three categories (Figure 1): VLPs: Mimicking the natural viral capsid structure, VLPs possess strong immunogenicity as well as the capability for nucleic acid encapsulation and delivery. Natural self-assembling protein nanoparticles: Examples include bacterial microcompartments, ferritin, lumazine synthase, and encapsulin, which perform diverse physiological functions, including catalysis, storage, metabolism, and homeostasis. Artificially designed protein nanoparticles: Constructed through computational design that emulates natural protein assembly principles, these synthetic systems possess specific structural and functional properties [5,6].

Both the internal and external surfaces of SPNPs can be readily modified to display peptides or functional molecules, facilitating drug loading, targeted delivery, and controlled release. Compared with chemically synthesized or polymeric/lipid nanoparticles, SPNPs exhibit greater stability across a range of pH levels, temperature, and solvent conditions, and can be efficiently produced in diverse expression systems, including bacterial, yeast, plant, insect, and mammalian cells, as well as cell-free systems [7]. These features make SPNPs ideal platforms for vaccine development and immunotherapy. They can effectively induce immune responses, enhance drug delivery efficiency, and demonstrate significant potential in biomedical and material science applications.

## 2. VLPs in Vaccine Research

VLPs are biologically derived nanoparticles formed through the self-assembly of viral structural proteins. They resemble the outer shell of viruses but lack viral genomes, rendering them non-infectious [8]. VLPs typically adopt hollow polyhedral structures composed of protein subunits, and their highly ordered geometrical arrangement can effectively activate the immune system.

Currently, various VLPs from different sources, including animal viruses, bacteriophages, and plant viruses, have been widely studied and developed for vaccine production and other biomedical applications. Depending on viral complexity, VLPs may be composed of one or multiple structural proteins, exhibiting size, morphology, and immunogenicity similar to native viral particles. The capsid proteins of VLPs are highly versatile and can be modified on either the interior or exterior surfaces to adjust particle size, antigenicity, cellular targeting, and antigen-loading capacity, thereby expanding their applications in vaccines and other biomedical fields [9].

As a vaccine platform, VLPs possess nanoscale dimensions (20–200 nm) and highly repetitive antigen presentation patterns, allowing efficient drainage to lymph nodes—the key sites for immune activation—and promoting uptake by antigen-presenting cells (APCs). They exhibit strong immunogenicity. Furthermore, the unique geometry of VLPs can trigger Toll-like receptor (TLR) signaling pathways and promote B-cell receptor clustering, enhancing immune responses [10]. Consequently, VLPs can display epitopes in a multivalent and repetitive manner, effectively inducing both humoral (B-cell mediated) and cellular (T-cell mediated) immune responses [11].

### 2.1. Animal Virus-Derived VLPs

Among animal virus-derived VLPs, Hepatitis B virus (HBV) VLPs are the most representative. Their capsids consist of 240 surface antigen subunits, forming icosahedral (T = 4) nanoparticles with a diameter of approximately 35 nm [8]. Sun et al. used a plant expression system to produce chimeric DIII HBV core antigen VLPs, which elicited protective immunity in mice against lethal doses of West Nile virus [12].

Several VLP-based vaccines have been approved for human use, including CoVLP (SARS-CoV-2 VLPs vaccine) [13], Epaxal (Hepatitis A vaccine) [14], Engerix-B [15] and Sci-B-Vax [16] (Hepatitis B vaccines), Hecolin [17] (Hepatitis E vaccine, approved only in China), and Cervarix and Gardasil 9 [18] (human papillomavirus (HPV) vaccines). Influenza VLPs vaccines, which mimic native viral structures and effectively induce both B-cell and T-cell immune responses, have also been approved. HPV VLPs vaccines prevent multi-type HPV infections and have been commercialized [18].

VIMKUNYA™ is a recombinant Chikungunya virus VLPs vaccine with a virus-like particle structure that induces strong neutralizing antibodies in clinical trials and has been approved for individuals aged ≥ 12 years [19]. Additionally, three veterinary VLPs vaccines are commercially available: Porcilis PC^®^ [20], Ingelvac CircoFLEX^®^ [21], and foot-and-mouth disease virus VLPs vaccines [22].

Numerous VLPs vaccines remain under development for both human and veterinary applications [23]. Human vaccine research focuses on enteric viruses, respiratory viruses, arboviruses [24], and highly pathogenic viruses, including Ebola virus (Ebola) and human immunodeficiency virus (HIV). Veterinary vaccine research emphasizes porcine diseases (e.g., Porcine Reproductive and Respiratory Syndrome, Porcine Circovirus type 2, porcine parvovirus), avian diseases (e.g., Highly Pathogenic Avian Influenza, Newcastle disease), and ruminant diseases (e.g., Peste des Petits Ruminants, bluetongue). Research on multivalent VLPs vaccines is also advancing. For example, Garg et al. [25] developed a VLP-based multivalent vaccine capable of inducing immune responses against Zika virus, Chikungunya virus, Yellow fever virus, and Japanese encephalitis virus in Balb/c mice, demonstrating the broad applicability of VLPs platforms for multi-pathogen vaccine development.

### 2.2. Bacteriophage-Derived VLPs

Bacteriophages are viruses that specifically infect bacteria without targeting eukaryotic cells, offering unique safety advantages and enabling large-scale production. Common bacteriophage VLPs platforms include MS2, Qβ, P22, and AP205 coat proteins. MS2 VLPs are primarily utilized to display small-molecule antigens and have been extensively applied in vaccines targeting viruses, parasites, Chlamydia, and cancer [26,27]. Qβ bacteriophage is an RNA phage infecting Escherichia coli, assembles into 25 nm icosahedral capsids composed of 178 coat protein subunits. Qβ VLPs have been employed to present a peptide derived from angiotensin II receptor type 1, effectively lowering blood pressure in hypertensive mice and potentially preventing diabetic nephropathy [23,24]. P22 VLPs, derived from the P22 bacteriophage—a double-stranded DNA phage infecting Salmonella typhimurium—form T = 7 icosahedral structures composed of capsid protein gp5 and scaffold protein gp8 [28]. As a vaccine platform, P22 VLPs can encapsulate influenza nucleoprotein and hemagglutinin, inducing protection against multiple influenza strains. AP205 bacteriophage VLPs have attracted attention for their exceptional antigen-display capability. Conjugating host self-antigens to AP205 VLPs can induce robust immune responses, overcoming B-cell tolerance to self-antigens [29,30]. AP205 capsid proteins also serve as scaffolds for high-density antigen display, such as the trimeric HIV-1 envelope glycoprotein gp140 [31]. Conjugation of the SARS-CoV-2 receptor-binding domain (RBD) to AP205 CP3 has successfully elicited potent neutralizing antibodies [32], and this platform has progressed to clinical trials (NCT04839146) [33]. However, bacteriophage VLPs often exhibit limited structural stability and a tendency to aggregate, necessitating engineering modifications and optimization of storage conditions to enhance particle stability and dispersibility [34,35].

### 2.3. Plant Virus-Derived VLPs

Plant-derived virus-like particles (plant VLPs) and their derivatives have become important platform technologies in vaccine development due to their unique advantages. As some of the simplest virus nanoparticles, plant VLPs exhibit several key characteristics: (1) they can undergo post-translational modifications and proper assembly in eukaryotic expression systems; (2) they pose minimal risk of contamination from exogenous pathogens; and (3) they offer low-cost, scalable production potential. Currently, various plant viruses have been successfully utilized for VLPs vaccine development, including Tobacco mosaic virus (TMV), Cucumber mosaic virus (CMV), Alfalfa mosaic virus (AMV), and Potato virus Y (PVY) (Table 1). Plant virus-derived VLPs hold considerable promise for vaccine research. In infectious disease prevention, plant VLPs technology has demonstrated substantial application potential. For instance, an AMV-based VLPs vaccine displaying the Pfs25 protein of Plasmodium falciparum showed excellent safety and tolerability in Phase I clinical trials [36,37]. PVY VLPs presenting the HBV preS1 antigen elicited potent neutralizing antibodies [38], while CMV VLPs displaying the EDIII domain of dengue virus serotype 1 envelope protein exhibited potential protective effects [39]. Moreover, VLPs derived from Physalis mottle virus (PhMV) and Malva mosaic virus (MaMV) displaying influenza M2e peptides induced immune protection in mouse models [40,41].

In tumor immunotherapy, TMV-derived VLPs presenting cytotoxic T lymphocyte (CTL) peptides significantly enhance melanoma treatment efficacy [42,43]. Similarly, PhMV VLPs displaying HER2 peptides effectively inhibit tumor growth and prolong survival. In allergy research, PVY and CMV VLPs presenting the cat allergen Fel-d1 induced high-titer antibody responses, and plant virus vectors expressing IL-17 or IL-5 induced cytokine-specific antibodies, thereby mitigating allergic reactions [44,45]. In other disease models, CMV VLPs displaying nerve growth factor (NGF) significantly alleviated postoperative spontaneous pain in mice [46]. Collectively, plant-derived VLPs technology, with its high flexibility, safety, and scalability, shows broad application prospects in infectious disease prevention, tumor therapy, and the management of allergic diseases.

**Table 1:** Plant virus-derived VLPs [23].

VLPs Platform	Target Pathogen/Disease	Vaccine Antigen/Epitope	Expression System	Reference
Alfalfa mosaic virus (AWV)	*Plasmodium* spp.	Pfs25	Eukaryotic (Tobacco plant)	[36,37]
Papaya mosaic virus (PapMV)	Hepatitis C virus (HCV)	E2 antigenic epitope	Prokaryotic (*E. coli*)	[47]
Cowpea mosaic virus (CMV)	Dengue virus	Domain III of envelope protein type 1 (DV1-EDIII)	Prokaryotic (*E. coli*)	[39]
Physalis mosaic virus (PhMV)	Influenza A virus	M2e peptide	Prokaryotic (*E. coli*)	[41]
Maize mosaic virus (MaMV)	H3N8 influenza virus	M2e peptide	Prokaryotic (*E. coli*)	[40]
Cowpea chlorotic mottle virus (CCMV)	Clostridium tetani	Tetanus toxin epitope (TT830–843)	Prokaryotic (*E. coli*)	[48]
	Group B Streptococcus type III	Capsular polysaccharide S9 peptide	Eukaryotic (*Pichia pastoris*)	[44]
Peanut yellow mosaic virus (PYV)	Hepatitis B virus (HBV)	preS1 domain	Prokaryotic (*E. coli*)	[38]
	Felis catus mite	Fel d 1 allergen	Prokaryotic (*E. coli*)	[49]
Cowpea mosaic virus (CMV)	Psoriasis/Alzheimer’s disease/Cat allergy	IL-17/β-amyloid peptide/Fel d 1	Prokaryotic (*E. coli*)	[44]
Tobacco mosaic virus (TMV)	Rabbit papillomavirus (CRPV and ROPV)	L2 capsid epitope	Prokaryotic (*E. coli*)	[50]
Physalis mosaic virus (PhMV)	Breast cancer	CH401 peptide derived from HER2	Prokaryotic (*E. coli*)	[51]
Turnip mosaic virus (TuMV)	Allergy	Prup3 allergen	Eukaryotic (Tobacco plant)	[52]
Cowpea mosaic virus (CMV)	Allergy	IgE-Fc fragment	Eukaryotic (HEK293F cells)	[53]

VLPs self-assemble into particulate structures directly derived from viral antigens, serving as efficient antigen presentation platforms that effectively enhance humoral immune responses. However, large-scale production of VLP-based vaccines remains challenging due to multiple technical and manufacturing limitations. Firstly, low expression yields significantly hinder industrial-scale production. In addition, contaminants derived from host cells require complex and costly purification procedures. Some VLPs must undergo in vitro disassembly and reassembly steps to improve structural stability [54]. Residual host-cell DNA/RNA present during this process can alter VLPs symmetry and increase particle polydispersity; pre-reassembly nuclease treatment is recommended to ensure consistent quality [55,56]. Moreover, the absence of viral genomes may further compromise particle integrity [57]. Enveloped VLPs rely on eukaryotic expression systems to acquire lipid bilayer structures, thereby increasing production complexity [58].

These challenges collectively limit the widespread application of VLPs in vaccine development. Nevertheless, recent advances in plant synthetic biology, molecular design, and vaccine engineering are gradually addressing these limitations.

Despite their strong immunogenicity, VLPs still exhibit inherent drawbacks such as complex manufacturing, potential pre-existing antiviral immunity, and difficulties in large-scale production. In contrast, natural protein nanoparticles—such as ferritin and encapsulin—offer more straightforward engineering, superior biocompatibility, and versatile antigen display capabilities. Therefore, while VLPs have laid a solid foundation for particulate vaccine development, natural protein nanoparticles represent a promising next-generation platforms for safe, efficient, and flexible antigen delivery.

## 3. Natural Protein Nanoparticles in Vaccine Research

Protein nanoparticles naturally present in organisms possess uniform size, defined structure, biodegradability, and facile modifiability, making them highly promising for vaccine research. Compared to VLPs, natural protein nanoparticles are widely sourced, structurally stable, and more controllable, allowing them to accommodate diverse antigen delivery strategies. While VLPs rely on viral capsid proteins to self-assemble into virus-like particles and exhibit excellent immunogenicity, they generally suffer from poor stability, limited adaptability to antigen size and type, complex production processes, and potential immune interference [59].

In contrast, natural protein nanoparticles can be precisely engineered through genetic and chemical modifications to control antigen presentation, enhancing immunogenicity while avoiding potential side effects associated with VLPs, such as inflammatory responses, immune interference due to pre-existing vector immunity, and production-related contaminants [60,61]. Currently, ferritin, encapsulin, lumazine synthase (LuS) [62], and the E2 component of pyruvate dehydrogenase complex (E2 nanocage) are widely studied natural protein nanoparticles for vaccines development, with applications in viral, bacterial, parasitic, and tumor vaccine.

### 3.1. Ferritin in Vaccine Research [63]

Ferritin is composed of 24 subunits that self-assemble into a protein shell surrounding an iron core, each ferritin core is capable of storing approximately 4500 iron atoms. Under artificial conditions, ferritin forms a hollow cage structure with an outer diameter of 12 nm and an inner diameter of 8 nm. Ferritin exhibits excellent self-assembly capability, high thermal stability, and is compatible with various preparation and modification techniques. Due to its high biocompatibility, stability, and ability to present multivalent or multiple antigens via its threefold symmetry axes, ferritin has been widely applied in vaccine research.

Ferritin is composed of 24 subunits that self-assemble into a protein shell surrounding an iron core. Each ferritin core is of storing approximately 4500 iron atoms. Under artificial conditions, ferritin forms a hollow cage structure with an outer diameter of 12 nm and an inner diameter of 8 nm. Ferritin exhibits excellent self-assembly capability and high thermal stability and is compatible with various preparation and modification techniques. Wild-type ferritin typically exhibits a melting temperature (Tm) of 80–85 °C, with certain variants up to 115 °C [64]. For example, the human cytomegalovirus (HCMV) gB vaccine antigen trAD5 has a Tm of approximately 45 °C, whereas its conjugation with ferritin yields trAD5-ferritin nanoparticles with a markedly improved Tm of approximately 58 °C [65]. In addition to thermal stabilization, ferritin can display nanobodies to significantly enhance binding affinity and extend in vivo half-life [66], further supporting ferritin as a multifunctional vaccine and antibody delivery platform.

Due to its high biocompatibility, stability, and the ability to present multivalent or multiple antigens using its threefold symmetry axes, ferritin has been widely applied in vaccine research. Ferritin nanoparticles have been explored for vaccines targeting infectious viruses, parasites, tumors, and bacteria [67]. For example, rabies is a zoonotic disease causing severe neurological disorders with nearly 100% mortality. Fu et al. used an innovative autophagy (Fagy) tag delivery platform to display optimized glycoprotein domain III (GDIII) on ferritin nanoparticles. The resulting GDIII-ferritin nanoparticle vaccine demonstrated enhanced antigen stability and elicited robust and broad humoral immune responses, as well as Th1-biased CD4+ T-cell responses. A single dose provided complete protection against rabies virus challenge in mice, highlighting its immunological advantages and application potential [68].

Additionally, ferritin nanoparticles designed using *Helicobacter pylori* ferritin have been applied to vaccines for SARS-CoV-2 and influenza viruses, achieving protection against single strains and generating broadly protective antibodies against multiple variants [69]. Ferritin nanoparticle vaccines have also been applied to other human viruses, including HIV [70], hepatitis B virus [71], Nipah virus [72], herpesvirus [73], and animal viruses such as porcine epidemic diarrhea virus [74], porcine reproductive and respiratory syndrome virus [75], foot-and-mouth disease virus [76], and African swine fever virus [77]. Beyond viral vaccines, ferritin nanoparticles are being investigated for tumor [78], malaria [79], antibiotic-resistant bacteria (e.g., Pseudomonas aeruginosa) [80], and chronic disease (hypercholesterolemia) [81] vaccines.

To date, dozens of ferritin nanoparticle vaccines have been studied, with six entering Phase I clinical trials [81]. Clinical studies of influenza ferritin nanoparticle vaccines have demonstrated safety and sustained induction of cross-neutralizing antibodies. In Phase I trials of SARS-CoV-2 vaccines, ferritin nanoparticle vaccines showed good tolerability and elicited potent, durable neutralizing antibodies against multiple SARS-CoV-2 variants [82]. Encouragingly, HIV ferritin nanoparticle vaccines have also entered clinical trials, demonstrating that ferritin nanoparticles serve as a versatile platform for vaccine development across various diseases.

### 3.2. Encapsulin in Vaccine Research

Encapsulin is a class of self-assembling icosahedral protein nanoparticles derived from prokaryotes, characterized by a unique spherical hollow structure that can be modified both internally and on the outer surface modifications [83,84]. This structure allows the encapsulation of diverse protein cargos and the display of various protein ligands on its surface. Encapsulins were first discovered in 1994 in the supernatant of Lactobacillus acidophilus M18, exhibiting broad-spectrum antibacterial activity [85]. Structurally, encapsulin nanoparticles are composed of subunits similar to viral capsid proteins and resemble the HK97 bacteriophage capsid [86].

Self-assembled encapsulin particles primarily exist in three types: T = 1 (24 nm), T = 3 (32 nm), and T = 4 (42 nm), each exhibiting differential stability against denaturants, extreme pH, and high temperatures [86]. Among them, T = 1 particles show strong resistance to extreme pH, denaturants, heat, and mechanical stress [87]. Natural encapsulins can encapsulate ferritin-like proteins or dye-decolorizing peroxidases, relying on C-terminal cargo-loading peptides (CLP) for specific and efficient cargo encapsulation [88].

Currently, encapsulin nanoparticles can be engineered as modular platforms through genetic modifications. Using CLPs and SpyTag/SpyCatcher technology, protein cargos can be encapsulated, and protein ligands displayed on the surface. The highly repetitive surface of encapsulin allows effective antigen epitope presentation. For example, displaying 60 copies of the Epstein–Barr virus gp350 D123 domain induced strong neutralizing antibody responses in mice and non-human primates [89]. Similarly, vaccines prepared by conjugating encapsulin with mRBD elicited potent neutralizing antibodies [90] and demonstrated excellent thermal stability, suitable for regions with limited cold-chain infrastructure. In animal disease vaccines, encapsulin displaying African swine fever virus antigens significantly enhanced antigen affinity and immunogenicity [84,91]. Encapsulin-based platforms have also shown high thermal stability in SARS-CoV-2 vaccine studies [90].

### 3.3. Lumazine Synthase in Vaccine Research

Lumazine synthase (LuS) is an enzyme complex involved in riboflavin (vitamin B2) biosynthesis, composed of LS subunits forming a shell and riboflavin synthase subunits forming the core. In the absence of riboflavin synthase, some prokaryotic LuS proteins, such as those from *Bacillus subtilis* and *Aquafex aeolicus*, can self-assemble into icosahedral (T = 1) nanoparticles composed of 60 identical subunits, with an outer diameter of approximately 15 nm and an inner diameter of about 9 nm [92]. Recent reviews have systematically summarized the morphology and functionalization of spherical LS nanoparticles [93]. By appropriate variation of amino acid sequences, LS can form different oligomeric states, including pentamers, decamers, 60-mer (T = 1 icosahedron), 180-mer (T = 3 icosahedron), 240-mer (T = 4 icosahedron), and even larger structures [94,95]. Among them, the T = 1 LS, composed of 60 identical subunits forming an icosahedron, has recently emerged as an attractive nanoparticle for multivalent antigen display [96]. Both the N- and C-termini of LS monomers are exposed outward, providing excellent conformational stability, making it a widely used platform for antigen presentation. For example, Li et al. used orthogonal dual-tag peptides to uniformly attach target antigens of Chikungunya virus and Zika virus on the LS surface, producing a bivalent nanoparticle vaccine [97]. Additionally, fusion of influenza matrix protein 2 (M2e) to the LS surface significantly enhanced immunogenicity and achieved 100% protection in mouse models [98]. LS-based HIV vaccines have also elicited robust T-cell immune responses in human trials [99].

### 3.4. Pyruvate Dehydrogenase E2 in Vaccine Research

Self-assembling E2 nanoparticles are derived from the E2 subunit of the pyruvate dehydrogenase complex from *Geobacillus stearothermophilus*. They self-assemble into highly symmetric 60-subunit dodecahedral nanoparticles with icosahedral symmetry (T = 1), an outer diameter of approximately 24 nm, an inner diameter of about 18 nm, and 12 surface pores each measuring ~5 nm [100]. The scaffold has three interfaces—the internal cavity, subunit-subunit interface, and outer surface—allowing molecular modifications for site-specific functionalization. Engineered E2 scaffolds exhibit exceptional thermal stability, unfolding only above 80 °C, making them suitable for high-temperature applications such as industrial enzyme immobilization or reactions [101].

E2 nanoparticles function as antigen presentation platforms by fusing or chemically conjugating exogenous peptides (e.g., viral antigens) to the surface, forming repetitive antigen arrays that significantly enhance immunogenicity. For instance, HIV surface antigens (gp120/gp140) fused to the N-terminus of E2 nanoparticles effectively activated B cells to produce high-titer antibodies [102]. Similar designs have been applied to display SARS-CoV-2 spike protein trimers, mimicking the native viral structure to induce neutralizing antibodies. E2 nanoparticles can also be used for drug-targeted delivery, with their hollow cavity and pores capable of encapsulating drugs or biomolecules. For example, the FDA Fast Track-designated CNP-104 nanoparticles encapsulate PDC-E2 antigens for treating autoimmune liver disease (primary biliary cholangitis), modulating immune responses by simulating apoptotic clearance, and have entered clinical trials (NCT05104853) [103]. E2 nanoparticles exhibit favorable biocompatibility and biodegradability, making them safe carriers suitable for delivering nucleic acids, proteins, or small molecules.

Beyond these, other natural self-assembling proteins, including Vault proteins, heat-shock proteins, and Dps, have emerged as promising nanoparticle carriers for future vaccine development [104,105,106,107,108]. Recent studies have also explored self-assembling CsgA (curli-specific gene A from E. coli) and its truncated variant R4R5CsgA as antigen delivery platforms, which can be genetically engineered to form self-adjuvanted nanofilaments that induce potent immune responses [109,110]. Although natural protein nanoparticles are diverse, biocompatible, and capable of self-assembly, they may induce some degree of non-specific immune responses when used as antigen carriers, potentially interfering with target antigen recognition [111,112]. Moreover, there is currently a lack of systematic scientific guidance for selecting nanoparticle types, and strategies for antigen display and loading require further optimization to achieve structural compatibility between the antigen and carrier while maximizing immunogenicity and minimizing non-specific immune activation. For instance, on T = 1 icosahedral scaffolds such as lumazine synthase (LuS) or encapsulin, displaying large antigens at high density is often hindered by steric crowding, which prevents full occupancy of all 60 subunits. Nevertheless, functional immunogenicity does not necessitate complete occupancy, and this challenge can be effectively mitigated by using longer flexible linkers, mosaic display of different antigens, or truncating the antigen to its minimal immunogenic domain [113,114,115].

## 4. Artificially Designed Protein Nanoparticles in Vaccine Research

Natural protein nanoparticles typically exhibit polyhedral geometric symmetry, and their outer surfaces, internal cavities, and subunit interfaces can be modified, offering remarkable versatility. Researchers have designed artificial protein nanoparticles based on the self-assembly principles of natural protein particles, often using symmetry-based fusion strategies. With the rapid development of computational technologies, computationally optimized and simulation-designed protein nanoparticles have become a reality. These nanoparticles include both naturally existing protein structures and newly designed synthetic structures.

Padilla et al. applied this strategy to construct tetrahedral protein nanoparticles [116]. Subsequently, King et al. used natural trimeric protein units to successfully create two types of artificial protein nanoparticles [117]. Zhang et al. engineered a 16-mer protein nanoparticle by inserting seven amino acid residues into ferritin [118]. Stupka et al. designed a TRAP cage using a 12-mer TRAP ring, demonstrating excellent thermal and alkali stability [119]. Artificially designed protein nanoparticles have achieved significant progress in vaccine research (Table 2). Hsia et al. introduced mutations at two surface cysteine residues of Thermotoga maritima aldolase I301, developing the Mi3 platform [120,121]. This platform self-assembles into a stable icosahedral structure of 60 subunits, exhibiting high particle stability and uniformity while allowing antigen fusion for vaccine development. Bruun et al. fused the SARS-CoV-2 receptor-binding domain (RBD) to Mi3 particles, inducing strong neutralizing antibody responses in mice and pigs, outperforming convalescent human serum [121]. Kang et al. further confirmed the immunological advantage of RBD-conjugated Mi3 nanoparticles [122].

Marcandalli et al. designed the I53-50 nanoparticle, which is composed of 20 trimers (I53-50A.4.1pt) and 12 pentamers (I53-50B.4PT1), forming a stable icosahedral structure [123]. This nanoparticle has been applied in SARS-CoV-2 [124], respiratory syncytial virus (RSV), and Epstein–Barr virus (EBV) vaccine research.

Yin-Feng Kang further developed a “Mosaic” quadrivalent SARS-CoV-2 protein nanoparticle vaccine based on I53-50. This vaccine is capable of inducing broad neutralizing antibodies against multiple SARS-CoV-2 variants and other coronaviruses in mice and cynomolgus monkeys, providing robust protection [122].

In parallel, researchers have developed the I3-01 nanoparticle as an additional highly stable dodecahedron platform. I3-01 is composed of 60 subunits assembled from trimeric building blocks, exhibiting excellent thermostability and structural uniformity, which makes it well-suited for antigen display and vaccine development [125,126,127].

Artificially designed protein nanocages, such as Mi3 and I53-50, have emerged as representative platforms in vaccine research. With rapid progress in artificial intelligence and computational protein design, the development of novel artificial protein nanoparticles is expected to accelerate in the near future [6]. For example, Neil P. King et al. employed computer-aided design to engineer multi-component, bifunctional protein nanomaterials. These nanomaterials feature two distinct and independently functionalized surfaces, enabling broad applications in biomedical fields [128]. Similarly, Professor Elliot L. Chaikof’s team developed the ENTER (Elastin-based Nanoparticles for Therapeutic Delivery) system, which efficiently delivers nucleic acids, proteins, and gene-editing tools into the cytoplasm [129].

**Table 2:** Application of Artificially Designed and Synthesized Protein Nanocages in Vaccine Research.

Nanoparticle Platform	Target Pathogen/ Antigen	Immunogenicity or Detection Performance	References
Mi3	SARS-CoV-2 (RBD)	Induced broad-spectrum neutralizing antibodies effective against multiple variants	[130,131]
	CSFV (E2)	Increased antibody titers tenfold and enhanced cross-genotype protection.	[132]
	Influenza A (NA)	Elicited higher antibody titers and protection against H1N1 and H3N2	[133]
	H9N2 (HA1)	Provided effective protection against lethal challenges from multiple H9N2 strains	[134]
	SARS-CoV-2	Enhanced neutralization against wild-type and variant strains	[135]
	PEDV (S1)	Promoted stronger humoral and cellular immune responses	[136]
	Aflatoxin B1/Ochratoxin A nanobodies	Enabled highly sensitive detection of both mycotoxins	[137]
I53-50	Respiratory syncytial virus (DS-Cav1/Sc9-10)	Conjugating RSV pre-fusion F to NPM significantly enhanced its immunogenicity, stability, and bioactivity compared to preF displayed on the I53-50 carrier	[138]
	SARS-CoV-2 (RBD)	Elicited strong neutralizing antibodies and effective protection	[122,139]
	RSV (F glycoprotein)	Induced tenfold higher neutralizing antibody levels than DS-Cav1	[123]
	EBV (gB)	Induced potent and durable neutralizing antibodies.	[140]
	Epstein–Barr (gb + gHgL)	Enhanced vaccine immunogenicity and neutralizing antibody induction	[141]
	Influenza (HA)	Generated broad cross-protective neutralizing antibodies	[142]
	EBOV and SUDV (GP antigens)	Provides broad protection against both Zaire and Sudan strains in mouse and guinea pig models	[143]
	*P. falciparum* (HLA class I-restricted epitopes)	elicited increased IFN-γ T-cell Responses against the inserted epitopes and significantly higher antibody	[144]
I3-01	Filovirus (glycoproteins)	Antibody responses induced by filovirus GP trimers and SApNPs bearing wildtype or modified glycans are assessed in mice	[125]
	EBV (gp350)	Elicited higher titers of total IgG and neutralizing antibodies	[126]
	SARS-CoV-2 (RBD)	Enhanced SARS-CoV-2 clearance from the nose and lungs of Syrian hamsters	[127]

Artificially designed protein nanoparticles are constructed either based on natural protein structural templates or entirely novel structural units. Computer-aided design (CAD) and molecular dynamics (MD) simulations are used to create self-assembling nanostructures [117,145,146]. This strategy enables precise control over protein spatial conformation, subunit interaction interfaces, and surface functionalization sites at atomic or sub-nanometer resolution. Consequently, it allows rational regulation of particle size, symmetry, assembly pathways, and antigen presentation modes.

During the design process, researchers can incorporate specific functional modules according to immunological requirements, such as antigenic epitope display regions, receptor-binding domains, or self-adjuvanting peptide motifs. This enhancing the targeting ability and immune-stimulatory capacity of the nanoparticles as vaccine carriers.

Although artificially designed protein nanoparticles offer significant advantages in structural predictability and functional customizability, several challenges remain. Structural models generated by computational simulations require experimental validation through heterologous expression and biochemical analyses to confirm assembly stability. Additionally, it is necessary to evaluate the conformational integrity and immune-enhancing effects after antigen conjugation. Different expression systems (e.g., *E. coli*, yeast, or mammalian cells) may affect assembly efficiency and structural fidelity. Moreover, the experimental success rate from computational design to successfully assembled particles is approximately 30%, and the “dark matter” of failed designs (e.g., poor expression, incorrect oligomerization, or polydisperse aggregation) remains severely underreported in the literature [147]. These issues impose higher demands on experimental verification and optimization for practical applications.

## 5. Summary and Outlook

SPNPs have demonstrated remarkable advantages in vaccine development, particularly against SARS-CoV-2, influenza virus, emerging pathogens, and other major infectious diseases, exhibiting excellent antigen delivery and immune-enhancing capabilities [141,148]. They can be recombinantly expressed in both prokaryotic and eukaryotic systems with controllable parameters, providing a robust technological foundation for the development of safe, efficient, and scalable next-generation vaccines. Continuous improvements in delivery platform design, adjuvant coordination, and structural optimization strategies have positioned SPNPs as a key innovative platform connecting basic research and clinical application.

Based on their origin and construction, SPNPs can be classified into three categories: VLPs, natural protein nanoparticles, and artificially designed protein nanoparticles. VLPs possess highly virus-mimetic structures, effectively inducing humoral and cellular immunity. However, their production is complex, and stability and cost persist. Natural protein nanoparticles are easier to produce, exhibit excellent biocompatibility, and allow controllable self-assembly, making them suitable for multivalent antigen display. Nevertheless, their loading capacity and immunomodulatory precision remain limited. Artificially designed protein nanoparticles enable precise antigen presentation, targeted delivery, and tunable immune regulation through computational design and site-specific engineering. They provide high structural versatility but require sophisticated design platforms and extensive validation before clinical translation. Notably, SPNPs in the 20–200 nm size range with repetitive antigen display possess intrinsic adjuvanticity; however, external adjuvants remain necessary when particle size falls outside this range, when antigens are poorly immunogenic, when strong Th1-biased cellular immunity is required, or for mucosal immunization [11,149,150].

Advances in bioengineering further expanded the functional expansion of SPNPs. In-depth studies on self-assembly rules, spatial conformation, and cargo-loading mechanisms can significantly improve antigen delivery efficiency and immune activation. In platform design, physicochemical parameters such as particle size, charge, and surface modification should be systematically evaluated for their effects on in vivo distribution and immune pathways, while considering storage stability and long-term immunogenicity. Recently, nanoparticle-based delivery systems have shown promising outcomes in both animal and human vaccine studies, highlighting their broad applicability and translational potential [151].

Moreover, mRNA-driven computationally designed protein nanoparticle vaccines combine the rapid production advantages of mRNA technology with the high immunogenicity of self-assembling protein particles, representing a key development direction for SPNP platforms [152].

However, the broad application of SPNPs still faces several challenges: Insufficient directional control of antigen presentation may limit epitope exposure; the co-loading of multiple antigens can cause steric hindrance, which affects particle stability; and the risks of immune evasion or tolerance must be considered.

In terms of production, different expression systems may have limitations in glycosylation, folding, and purification efficiency.

Future research should focus on:

(1) Mechanistic elucidation—systematically clarifying the molecular interactions between different protein nanoparticles and the immune system;
(2) Structural optimization—leveraging structural biology and computational design for multivalent antigen co-loading, precise targeting, and controlled release;
(3) Safety and protective efficacy—investigating biosafety and the mechanisms of durable protection;
(4) Broad-spectrum applications—extending the platform use to emerging pathogens, drug-resistant bacteria, and tumor immunotherapy [153].

With the deep integration of materials science, structural biology, immunology, and artificial intelligence, protein nanoparticles are expected to achieve higher levels of precision, intelligence, and personalization. Through multidisciplinary innovation, SPNPs will become an essential support system for next-generation vaccine design, driving vaccine science into an era centered on structural design and immune modulation.

## Data Availability

No new data were created or analyzed in this study. Data sharing is not applicable to this article.

## Abbreviations

The following abbreviations are used in this manuscript:

SPNPs	Self-assembling protein nanoparticles
SARS-CoV-2	Severe Acute Respiratory Syndrome Coronavirus 2
RBD	Receptor-binding domain
VLPs	Virus-like particles
RSV	Respiratory syncytial virus
EBV	Epstein–Barr Virus
